# When social media becomes medicine: clinician-moderated online consultations as an informal healthcare access point

**DOI:** 10.1186/s13584-026-00777-w

**Published:** 2026-09-01

**Authors:** Inna Bleicher, Maya Peled-Raz, Sun Bleicher, Sharon Tzemah

**Affiliations:** 1 The Ruth and Bruce Rappaport Faculty of Medicine, Technion - Israel Institute of Technology, Haifa, Israel; 2 Department of Obstetrics and Gynecology, Bnai -Zion Medical Center, Haifa, Israel; 3 School of Public Health, University of Haifa, Haifa, Israel; 4 The Department of Psychology, Bar-Ilan University, Ramat-Gan, Israel; 5 Clalit Health Services, Post-Discharge Care Unit, Home Hospice Department, Haifa, Israel

**Keywords:** Digital health, Social media, Online medical consultation, Health literacy, Healthcare accessibility, Healthcare access

## Abstract

**Background:**

Social media has evolved significantly as a platform for healthcare communication, education, and consultation, especially following the COVID-19 pandemic. While these platforms are increasingly used for medical advice, they operate largely outside formal health policy frameworks, creating a growing “informal layer” of healthcare.

**Methods:**

A cross-sectional observational study was conducted using an anonymous online questionnaire distributed to the “*Ask an M.D*.” group members from April to July 2023. Demographic characteristics, consultation patterns, motivations for using the platform, and comparisons between users from central and peripheral regions of Israel were analyzed, to identify potential disparities in digital health access.

**Results:**

Of approximately 19,000 members who viewed the survey, 1,508 responded (8%). Respondents were predominantly female (85%), with a mean age of 38.5 years, and 61.5% reported higher education. Common reasons for using the group included seeking a second opinion (36%), delays in obtaining a doctor’s appointment (34%), and urgent health concerns (28%). Consultation topics most often involved gynecology and obstetrics (19%), pediatrics (15%), and dermatology (10.5%). Peripheral respondents were underrepresented relative to their proportion in the wider population. Within the respondent sample, central and peripheral participants showed broadly similar reported patterns of platform use.

**Conclusions:**

Clinician-moderated social media groups such as “Ask an M.D.” may serve as a supplementary source of health information and informal consultation for some users, particularly when immediate formal care is less accessible. The lower representation of peripheral respondents in this survey should be interpreted cautiously and may reflect differences in survey exposure, response propensity, platform reach, or other factors. Because this study did not assess clinical outcomes, it cannot determine whether participation in the platform improves care, safety, or health equity.

**Supplementary Information:**

The online version contains supplementary material available at https://doi.org/10.1186/s13584-026-00777-w.

## Background

In recent years, social media has evolved beyond its initial role as a networking tool, becoming a significant platform for health-related communication, education, and medical consultation. Digital platforms such as Facebook, Twitter, and WhatsApp allow users to seek medical advice, share experiences, and access health information outside the traditional healthcare system. This transformation was accelerated by the COVID-19 pandemic, which underscored the potential of online spaces to bridge healthcare accessibility gaps and facilitate real-time medical discourse [1–3].

Numerous studies have explored how healthcare professionals use social networking sites (SNSs) for professional communication, education, and collaboration. Facebook groups, in particular, have been used by clinicians to crowdsource opinions, share resources, and engage in virtual communities of practice, especially during the COVID-19 pandemic [4]). Systematic reviews have documented the role of SNSs in enhancing peer-to-peer interaction, disseminating clinical knowledge, and supporting evidence-informed practice [5, 6].

Increased use of these platforms has been accompanied by concerns regarding their potential risks. Studies have highlighted that information shared in social media settings may often be inaccurate or incomplete, as posts are not subject to formal peer review or professional verification prior to dissemination, potentially leading to misinformation and inappropriate health decisions [7, 8]. Additional concerns include breaches of patient privacy and confidentiality when sensitive information is disclosed in online forums [7, 8], and the absence of regulatory oversight to ensure the qualifications and accountability of those providing medical advice [8, 9]. Such interactions may also blur professional boundaries between clinicians and patients, creating ethical dilemmas and potentially undermining trust in traditional clinical relationships [7].

These regulatory and ethical concerns have prompted the development of professional guidelines by several leading bodies – aimed at governing care professionals’ involvement in such platforms. The American Medical Association’s (AMA) 2012 guidelines [10], for example, emphasize the importance of preserving patient privacy, maintaining professional boundaries, and avoiding the dissemination of unverified medical information through social media channels [7].

Similarly, the American College of Surgeons (ACS) encourages surgeons to uphold professional standards when engaging online, noting that social media activity can affect the public’s trust in the medical profession [8, 11].

In Israel, the Israeli Medical Association (IMA) has issued guidelines cautioning physicians against giving medical advice on social media without a proper clinical context, citing concerns about liability, confidentiality, and the absence of direct examination [12]. Yet, despite these guidelines, the phenomenon persists and grows, creating a vital “informal layer” of healthcare operating alongside the formal one.

On the user side of the equation, Gallardo and colleagues systematically reviewed the drivers and patterns of online health information seeking in social media contexts, emphasizing users’ motivations such as accessibility, speed, anonymity, and the availability of peer experiences [13]. These findings are echoed by Farsi et al., who provide a comprehensive overview of patients’ use of social media across five domains: access to health information, telemedicine, provider selection, peer support, and health behavior change [9].

These motivations are further shaped by structural inefficiencies within the formal health policy framework, such as users’ dissatisfaction with traditional health systems, long waiting times, geographic or economic barriers, and the need for quick and convenient access to health-related advice. Moorhead et al. emphasized that users are drawn to social media for health-related purposes primarily to access credible health information, receive emotional support, and share experiences, all within platforms that offer immediacy and community validation [14]. Almost a decade later, Gabarron et al. similarly describe social media as a health communication channel that enables users to bypass formal healthcare limitations by sharing experiences and obtaining advice, especially in regions or populations underserved by traditional health services [15].

Users often report turning to social media as a first or complementary step before seeking formal medical care, especially in situations of uncertainty, embarrassment, or limited access to professional consultation [12, 16], highlighting a potential role for these platforms in enhancing health equity.

Among these access-related motivations, geographic and financial barriers are often cited. Social media offers an alternative pathway for individuals living in remote or underserved regions to consult healthcare professionals or obtain peer-based health advice [12, 13, 15]. Similarly, for users who lack health insurance or who face high out-of-pocket costs, online platforms can serve as a cost-free or low-cost entry point into health information and guidance [14]. This makes professionally moderated groups especially attractive to those facing structural barriers to care.

In terms of demographics, studies consistently show that women are more likely to seek and share health information on social media, particularly in relation to maternal and child health [17]. Younger adults (under 40) tend to be more active in using digital platforms for health purposes, although older users are increasingly engaged as well. Educational level and digital health literacy were also found to play important roles in the likelihood and nature of engagement [16].

While many users turn to platforms like Facebook and WhatsApp to share experiences and provide mutual emotional support, others actively seek professional advice from licensed clinicians operating in these digital spaces. Lupton’s research in maternal health highlights this distinction, showing that pregnant individuals often use social media both for peer reassurance and for targeted advice from health professionals, especially in the absence of accessible or satisfying formal healthcare interactions [17].

Despite the growing body of literature, much of the focus remains on social or peer-support health groups, with relatively little systematic attention to social media groups that are explicitly moderated by licensed professionals providing tailored medical guidance [15]. While the potential of digital health to bridge gaps is often touted, the rise of un-institutionalized, clinician-moderated platforms poses a unique challenge for health policy. Unlike formal telemedicine initiatives managed by health maintenance organizations (HMOs), these ‘grassroots’ platforms operate in a regulatory grey zone. They offer immediate access but lack integration with patient records and formal oversight. For policymakers, this raises critical questions: Does this phenomenon mitigate health disparities or exacerbate them by serving only the digitally literate? And should these platforms be viewed as a rogue element or a vital buffer for an overburdened system?

### Ask an M.D. as a case study

*“Ask an M.D.”* is Israel’s largest health-related Facebook group, which provides free, professional medical advice to the public. Established in 2018, the group experienced a sharp increase in membership during the Covid pandemic, growing from 5,000 members in September 2019 to 19,000 members in September 2020. Today, it serves over 52,000 members, with responses provided exclusively by healthcare professionals, including approximately 130 physicians and 50 pharmacists. This model offers timely, evidence-based guidance, supplementing traditional healthcare services and improving public access to reliable medical information.

The rise of digital health consultations on social media highlights both opportunities and challenges for policymakers. On the one hand, platforms like *“Ask an M.D.”* enable rapid knowledge dissemination and peer-to-peer medical support, helping individuals make informed decisions about their health. On the other hand, concerns regarding misinformation, privacy risks, and the quality of online medical advice remain significant challenges [15].

This study uses *“Ask an M.D.”* as a case study for an online medical consultation platform. Its objectives were threefold: (1) To describe the demographic characteristics of users engaging with the “Ask an M.D.” Facebook group; (2) To explore their motivations for seeking medical consultations through the platform; and (3) To compare usage patterns between users residing in central and peripheral regions of Israel, in order to assess potential disparities in digital health access and engagement. By examining engagement trends, demographic factors, and consultation motivations, this study aims to provide empirical data to inform health policy regarding the evolving role of social media in modern healthcare delivery, shifting the analytical lens from the professional to the public, illuminating the conditions under which individuals rely on these “informal” systems to bridge gaps in formal care.

## Methods

### Setting

We performed a cross-sectional observational study, in which a convenience sample of “Ask an M.D” group members were surveyed using an online anonymous questionnaire. The questionnaire was posted on the group’s main page accompanied by a forward explanation sheet. Data was collected between April- July 2023. This study was approved by the local institutional review board (# 132 − 23).

The study was designed in conjunction with STROBE [18] and the Checklist for Reporting Results of Internet E-Surveys (CHERRIES) guidelines [19].

Since no validated tools were identified for this topic, our questionnaire was developed relying on clinical expertise and consultation with selected group members.

The introduction included an explanation of the target population and the study rational and content. Participants were notified that the survey is anonymous, requires approximately 5 min for completion, and is entirely voluntary. Additionally, they were informed that the questionnaire results may be compiled and published as a scientific article. To ensure informed consent, respondents were instructed to access the questionnaire site only if they agreed to take part in the survey.

The questions were fixed, and adaptive questioning processes were used. The questionnaire was administered in Hebrew; the translated document is available as Supplement 1. Non-response options were not used, considering the noninvasive nature of the questioner. The responses to the survey were collected using Google forms. Cookies were not used, and IP addresses were not collected, thus unique visitor counts were not calculated. Yet, since the survey was distributed on a single specific Facebook page, view rates were available to the group admin (one of the authors). Following the survey completion, the data was downloaded and permanently deleted from Google forms.

In addition, anonymous aggregated administrative group data was collected, to characterize posts’ distribution over time, engagement of participants in posts written by others (via number of reactions) and members’ demographic characteristics.

### Participants and sample size

All “Ask an MD” group members were eligible to participate except those who were serving as consultants in the group (physicians, pharmacologists, and nurses) which were excluded. A convenience sample was used, aspiring to achieve at least 5% response rate of those who viewed the survey.

#### Data collection and variables

The questionnaire consisted of two parts: the first included a total of 6 demographic questions (age, gender, ethnicity, education, geographic location and health-funds membership). The second part included 10 multiple choice questions enquiring about participants’ experience in the group and the rational for using it. Participants were asked about their consultation topic (medical field of last consultation) scope of usage of group’s services (did the member ever post a consultation, number of consultations in the previous year), their reasons for using the group for consultation, satisfaction with response received and use of other information sources outside the group.

As part of the study design, we explored whether reported platform use differed between respondents residing in central and peripheral regions of Israel. Participants’ geographic locations were grouped as “Central,” “Peripheral,” or “Neither,” according to the Central Bureau of Statistics. For the subgroup comparison, respondents classified as “Neither” were not included in the central-versus-peripheral analysis. Because the survey was based on a convenience sample, these subgroup comparisons were considered exploratory and were interpreted cautiously.

The number of consultations in the past year was categorized into three ordered groups: no consultations, one or two consultations, and three or more consultations. Education levels were similarly collapsed from six categories into three: “high school education or lower,” “bachelor’s degree/professional training,” and “master’s/PhD.”

Analyses were primarily descriptive. Categorical variables are presented as counts and percentages, and continuous variables as mean ± standard deviation. Percentages were calculated using non-missing responses for each item; therefore, denominators vary across variables. Central-versus-peripheral comparisons were treated as exploratory subgroup descriptions within the respondent sample rather than as population-level estimates. To reduce overinterpretation of a convenience sample, the revised manuscript emphasizes descriptive patterns rather than statistical significance.

## Results

### Group administrative data

In the year preceding our data collection, the group’s page had a total of 8,445 posts, which generated approximately 34,646 comments and 52,576 reactions. Activity peaked on Thursdays and Fridays, especially after 8:00 PM.

Among the group members, 77% were female, 94% resided in urban areas, and 6% were located outside of Israel. Regarding age distribution, 46% were aged 25–34 years, and 29% were 35–44 years old.

### Study participants

The questionnaire was distributed to the entire population of the “Ask an MD” group, (approximately 50 k members) and viewed by approximately 19,000 members, of which 1508 members (8%) replied to the questionnaire (i.e., the sample).

### Descriptive statistics

Of the respondents, 85% were female, and 96.8$ were Jewish. Geographically, 34% resided in Israel’s central regions (“Center”), 18% in peripheral areas (“Periphery”), and 48% were categorized as “Neither.” The mean age was 38.5 years (range: 15–80 years), and 61.5% reported having higher education.

A majority of respondents (56%) had previously asked questions within the group, and nearly all (99%) indicated they regularly read consultations posted by other members. Among actively consulting participants, the annual number of questions ranged from one (41%) to more than ten (1%). Most questions related to gynecology and obstetrics (19%), pediatrics (15%), and dermatology (10.5%). Notably, 15% of consulting participants were unsure about which medical discipline their question pertained to.

The primary reasons cited for using the group included seeking a second opinion (36%), encountering delays in securing a doctor’s appointment (34%), and addressing urgent health concerns (28%). Only 7.4% of participants exclusively used “Ask M.D.” for social media-based medical consultations. Additional information sources commonly reported included Google (77%), health fund websites (57%), the Ministry of Health’s website (28%), and other social media groups (22%).

Of the 1,508 respondents, 726 participants classified as “Neither” central nor peripheral were excluded from the subgroup comparison. The descriptive central-versus-peripheral comparison therefore included 267 peripheral respondents and 515 central respondents. For selected descriptive data from this comparison sample, see Table 1.

**Table 1:** Selected descriptive comparison of respondents from peripheral and central regions

Characteristic	Periphery	Central
**Respondent characteristics**		
Women, %	84.6	84.6
Age, years, mean ± SD	34.6 ± 10.3	37.8 ± 11.7
Jewish ethnicity, %	94.0	96.9
**Education level, % among non-missing responses (n = 234; n = 470)**		
Secondary or less	37.2	31.9
Bachelor’s degree / professional training	41.5	44.5
Master’s / PhD	21.4	23.6
**Health insurance provider, % among non-missing responses (n = 233; n = 464)**		
Clalit	67.0	45.5
Maccabi	24.5	43.5
Meuhedet	4.3	8.2
Leumit	4.3	2.8
**Consultation discipline, % among respondents reporting a discipline (n = 121; n = 239)**		
Ob/Gyn	27.3	21.8
Pediatrics	16.5	18.4
Dermatology	13.2	13.4
ENT	7.4	5.0
Ophthalmology	2.5	2.5
Surgery	1.7	5.9
Orthopedics	3.3	9.6
Other disciplines	28.1	23.4
**Reason for posting, % among respondents reporting a reason (n = 107; n = 243)**		
Urgent health concern	20.6	21.0
Did not know which doctor to ask	29.0	31.7
Consulted a doctor and did not receive a response	7.5	7.4
Second opinion	3.7	5.8
Did not understand the doctor	2.8	3.7
Felt uncomfortable asking	10.3	7.4
Appointment too far away	26.2	23.0

Note. Values are percentages unless otherwise indicated. Periphery *n* = 267; central *n* = 515. Denominators vary across items because percentages were calculated using non-missing responses, and some items applied only to respondents who had posted a consultation. The table is presented descriptively; no p-values are shown

Among respondents classified as residing in central or peripheral regions, the overall pattern of reported platform use was broadly similar. Consultation disciplines and stated reasons for posting were descriptively comparable across the two groups. Peripheral respondents were underrepresented among survey respondents relative to their proportion in the general population; however, in a voluntary survey conducted within a single online group, that finding may reflect several explanations, including differences in survey exposure, response propensity, characteristics of the group membership, or platform reach.

## Discussion

Our study complements recent literature exploring patients’ health-seeking behaviors on social media. Farsi et al. [13] highlighted that patients frequently rely on platforms such as Facebook and WhatsApp for information, peer support, and informal medical advice – often before consulting health professionals. Yet, as their review emphasizes, few studies have examined user motivations in a structured empirical manner. Our data fill this empirical gap by offering quantitative insight into the specific circumstances under which users turn to clinician-moderated platforms, particularly when formal access to care is delayed.

“Ask an M.D.” platform serves as a valuable resource for Israeli users seeking medical information and consultations online. This study provides insights into user engagement patterns, demographic profiles, and motivations, emphasizing the comparison between users residing in central and peripheral regions.

### COVID-19 pandemic and digital health service utilization

The surge in membership and activity on “Ask an M.D.” during the COVID-19 pandemic underscores the role of these extraordinary circumstances in accelerating digital healthcare adoption. The platform’s sustained usage, even as pandemic restrictions have eased, suggests that this shift may represent a lasting change in health-seeking behaviors. Users now see platforms like “Ask an M.D.” as reliable, on-demand sources of medical information, which supplement traditional healthcare. We observed increased usage towards the end of the week, likely reflecting the limited availability of in-person healthcare services over weekends. This trend reinforces the role of online consultation as a convenient alternative for timely health advice during periods when traditional healthcare may be less accessible. Such usage patterns suggest that the platform not only fulfills a need for immediate consultation but also complements existing healthcare services during off-hours, providing reassurance and support to users when other options may be unavailable.

### Analysis of central vs. peripheral usage patterns

Only 18% of respondents resided in peripheral areas, which is lower than their approximate share in the broader Israeli population. This observation should be interpreted cautiously. In a voluntary survey distributed within a single online group, lower peripheral representation may reflect multiple factors, including differential exposure to the survey, differential willingness to respond, characteristics of the group’s membership, or differences in use of the platform itself. The present study cannot distinguish between these explanations.

Among respondents from central and peripheral regions who participated in the survey, reported patterns of use were broadly similar at a descriptive level. These subgroup findings should also be interpreted cautiously, as the survey relied on a convenience sample and was not designed to generate population-level estimates.

### Demographic insights and comparison with existing data

Our findings show higher platform usage among women, younger individuals, and those with higher levels of education. Although these finding correlate with some scoping literature [16, 20], they do not align with the recent Israeli national survey on digital health literacy survey [21] which found no significant differences in digital health literacy or in the use of digital health platforms between men and women. Moreover, and contrary to our findings, the HLS19-Isr survey observed no correlation between education level and digital health literacy, while finding that digital health literacy and digital health engagement are highly correlated. This contradicts the expectation – somewhat supported by our results – that higher education would predict greater engagement with digital health tools.

Additionally, while our study found that younger individuals were more likely to use digital health platforms, the HLS19-Isr survey identified only a weak negative correlation between age and digital health literacy, indicating that the impact of age on engagement with these tools is relatively small. These findings are not unique to Israel. The International Report of the European Health Literacy Population Survey 2019–2021 [22] also showed similar patterns across multiple European countries.

Taken together, these findings suggest that participation in clinician-moderated online consultation groups reflects more than demographic characteristics alone. However, because the present study did not directly measure digital health literacy, internet access, or structural barriers, such factors should be considered possible explanations rather than conclusions supported by the current data.

### User motivations and consultation trends

Our survey revealed that the primary motivations for using the platform include the need for a second opinion (36%), delays in obtaining doctor appointments (34%), and addressing urgent health concerns (28%). These motivations appeared similar in the descriptive subgroup review of central and peripheral respondents, suggesting that the platform was used in comparable ways by respondents from both regions. The high prevalence of gynecology and obstetrics queries is closely related to the platform’s user demographics, which are predominantly young women. In our cohort, 77% of users were female, and 46% were aged 25–34, a demographic likely to seek consultation on reproductive health. This pattern emphasizes that the nature of the topics discussed aligns with the characteristics and needs of the primary user base, reflecting both personal health priorities and access needs within this group. Additionally, about 15% of respondents were unsure of the specific medical discipline relevant to their question, highlighting the platform’s role in providing general guidance when users are uncertain about which healthcare specialty they should consult.

### Policy implications - informal digital health ecologies and regulatory frameworks

As shown in our findings, the “*Ask an M.D*.” Facebook group offers a unique hybrid model of digitally mediated medical interaction: it is publicly accessible, moderated by certified physicians, nurses, and pharmacists, and carefully bounded to avoid diagnostic or therapeutic recommendations when clinical context is lacking. Nevertheless, users often treat these exchanges as meaningful substitutes for clinical consultation—highlighting a broader phenomenon of non-institutional health-seeking behavior.

This phenomenon can be situated within what may be called “informal digital health ecologies”: decentralized, user-driven, and often semi-public environments in which health information, advice, and support are shared outside the structures of formal healthcare provision [15, 23]. Lupton’s qualitative research among pregnant women and new mothers shows that many users actively rely on social media groups, forums, and apps to supplement or even replace traditional medical encounters, especially when institutional care is perceived as slow, inaccessible, or emotionally lacking [17].

Our study reinforces this dynamic: the most frequent consultation topics in our sample were related to pregnancy and infant care—domains that are especially salient in informal digital contexts due to their urgency and emotional intensity [17]. In this light, the trust placed in Ask an M.D. appears to derive not only from the professional credentials of its moderators, but also from the immediacy, responsiveness, and tone of care conveyed within the group—qualities at times absent from institutional encounters.

Recognizing such platforms as part of broader informal digital health ecologies calls for a more nuanced policy perspective. While it is tempting to frame these interactions as inherently unregulated or ethically problematic, it is important to acknowledge both their limitations and their potential benefits. Professional and regulatory bodies should engage with the growing role of these platforms in the everyday health behaviors of the public, carefully weighing the risks of misinformation and lack of oversight against the unique opportunities they provide for enhancing access to health information and support.

While empirical data directly linking participation in online medical consultation groups like ‘Ask an M.D.’ to improved clinical health outcomes is currently limited, the potential benefits of such platforms as a formalized ‘informal’ healthcare layer cannot be overlooked. Our findings demonstrate that the motivations for seeking online advice, such as long waiting times and urgent health concerns, are consistent across both central and peripheral regions, suggesting a universal systemic need that transcends geographic boundaries. Specifically, our findings highlight their pivotal role as mechanisms for preliminary triage by guiding the 15% of users who are uncertain about which medical specialty to consult, thereby streamlining their entry into the formal system. Furthermore, by providing a safe and professional alternative for the 77% of users who already rely on unmonitored Google searches, these platforms play a crucial role in mitigating the risks of medical misinformation. Finally, they offer immediate professional validation by acting as a “buffer” during off-hours and weekends, which may simultaneously prevent unnecessary emergency room visits and encourage timely care for acute medical issues.

As clinician-moderated platforms become more regulated and integrated into professional frameworks, their utility as a vital healthcare tool is expected to grow. Developing clear guidelines for ethical, transparent, and effective participation by licensed professionals in such spaces could help maximize their benefits while minimizing potential harm. Policy response should extend beyond guideline development to include dissemination, implementation, monitoring, and mechanisms for addressing inappropriate use.

### Strengths and limitations

This study provides first of its kind valuable insights into the use of social media for medical consultations, using engagement within the *“Ask an M.D.”* Facebook group as a case study. The study’s strengths include its large sample size, and the use of real-world data from an active online health community.

This study has several limitations. The survey was based on a convenience sample of group members who chose to respond, which limits generalizability and requires cautious interpretation of subgroup comparisons. As in other voluntary online surveys, selection and response bias are possible and cannot be excluded. Although respondent characteristics were broadly similar to those of the group’s administrative profile, this does not eliminate the possibility of systematic differences between respondents and nonrespondents. In addition, the study relied on self-reported behaviors and did not assess clinical outcomes, safety, or downstream healthcare utilization. The findings should therefore be understood as descriptive of this respondent sample rather than as evidence that participation in the platform improves care or reduces inequities.

## Conclusions

Our findings underscore the pivotal role of clinician-moderated social media platforms, such as “Ask an M.D.“, in the contemporary healthcare landscape. These platforms have evolved beyond their initial function as networking tools, becoming integral components of public health-seeking behavior that offer immediate, professional validation outside traditional clinical settings.

This phenomenon warrants systematic policy attention. Rather than viewing these interactions as marginal or strictly private, health authorities should recognize them as a significant informal layer of the healthcare system. Developing professional frameworks to ensure safety, equity, and ethical management – alongside public education and clear guidelines for moderators – is essential. Such integration into broader health strategies will be key to harnessing the potential of social media consultations while safeguarding public health.

## Supplementary Information

Below is the link to the electronic supplementary material.


Supplementary Material 1


## Data Availability

The datasets generated and analyzed during the current study are available from the corresponding author upon reasonable request.
