# Supplementary material for: When social media becomes medicine: clinician-moderated online consultations as an informal healthcare access point
Source: Isr J Health Policy Res. 2026 Sep 1;15:35. doi: 10.1186/s13584-026-00777-w (PMC13531886; doi:10.1186/s13584-026-00777-w)
Supplement: Supplementary file 1 [file 13584_2026_777_MOESM1_ESM.docx]

**Supplement 1 – Survey Tool**

Dear group members,

We, the group admins, along with Dr. Maya Peled-Raz from the University of Haifa, invite you to participate in a short, anonymous survey regarding the use of this group.

To improve our service and better assist you, we would like to understand more about who uses this group, what their needs are, and whether we are successfully meeting these needs.

The survey is anonymous—no one will know if you participated or what your answers were. You are free to skip participation or withdraw from the survey at any time, but we would greatly appreciate it if you completed it. It will take no more than five minutes.

If the survey yields interesting results, we would endeavor to publish them so that others can learn from the findings.

Continuing to the survey implies your consent to participate.

Many thanks in advance,
Dr. Sharon Tzemah and Dr. Ina Bleicher
For questions, please contact Dr. Sharon Tzemah at: ….

**Questions**

1. **How did you hear about the group?**
   - Google search
   - Friend’s invitation
   - Saw someone’s post in my feed
   - Recommendation
   - Other: _______________
   - Don’t remember
2. **Have you ever posted a question in the group?**
   - Yes (Proceed to the next question)
   - No (Skip personal inquiry questions)
3. **If yes, how many posts with questions did you make in the past year?**
   - 1
   - 2
   - 3-5
   - 5-10
   - More than 10
4. **What was the subject of your question?**
   *(If unsure, feel free to select "Not sure")*
   - Gynecology (Women’s health and childbirth)
   - Dermatology
   - Orthopedics
   - Pediatrics
   - Surgery
   - ENT (Ear, Nose, Throat)
   - Ophthalmology
   - Other
   - Not sure

*If Gynecology, proceed to question 4a.*

4a. **What was the subject of your inquiry?**

- Questions about birth control and contraceptives
- Menstrual cycle-related questions
- Questions related to fertility treatments
- Questions about pregnancy tests
- Menopause-related questions
- General gynecology

*For all others/after proceed to question 5.*

5. **What led you to turn to this group for assistance?**

(Please select all that apply)

- It was urgent, and I couldn’t wait to speak to my doctor.
- The appointment was far away, so I consulted with the group in the meantime.
- I didn’t know which doctor to approach.
- I was uncomfortable asking my doctor about this.
- I asked my doctor but didn’t understand their explanation.
- I asked my doctor but didn’t receive a satisfactory answer.
- I wanted a second opinion.
- My post was about the doctor’s behavior.
- Other – Please specify: _______________

6. **7. Did you find the response you received in the group satisfactory?**
   - Yes, it was great, thank you.
   - I didn’t receive an answer in the group, but I was directed to where I should go for an answer.
   - I received an answer, but it didn’t help at all.
   - My post was published but didn’t receive a response.
   - My post wasn’t approved, and I didn’t understand why.
   - My post wasn’t approved, but I this was explaind by the admin, which also helped.
   - Other – Please specify: _______________
7. **Do you read posts uploaded by other group members?**
   - Yes
   - No
8. **Do you usually check if a question you’re interested in has already been asked?**
   - Yes
   - No
9. **Do you use additional online information sources besides this group?** (Please select all that apply... if none, leave blank)
   - HMO (Healthcare Maintenance Organization) websites
   - Ministry of Health website
   - General Google search
   - Other Facebook consulting groups – Please specify: _______________
   - Other: _______________

**Demographics**

- **Year of Birth**
- **Gender**
  - Male
  - Female
  - Other
- **HMO**
  - Clalit
  - Maccabi
  - Meuhedet
  - Leumit
- **Geographical Area**
  - Tel Aviv District
  - Center
  - Sharon
  - Shfela
  - Haifa
  - Jerusalem
  - Galilee
  - Golan
  - Negev and Eilat
  - Other
- **Type of Settlement**
  - Rural (Moshav, Village, Kibbutz...)
  - Urban
- **Years of Education**
  - Up to Grade 10
  - Completed high school without matriculation
  - Completed high school with matriculation
  - Vocational training
  - Bachelor’s degree
  - Master’s degree or higher
- **Ethnic/Religious Background**
  - Arab Muslim
  - Arab Christian
  - Non-Arab Christian
  - Jewish
  - Druze
  - Other
